# Methadone Inhibits Viral Restriction Factors and Facilitates HIV Infection in Macrophages

**DOI:** 10.3389/fimmu.2020.01253

**Published:** 2020-07-03

**Authors:** Mei-Rong Wang, Di-Di Wu, Fan Luo, Chao-Jie Zhong, Xin Wang, Ni Zhu, Ying-Jun Wu, Hai-Tao Hu, Yong Feng, Xu Wang, Hai-Rong Xiong, Wei Hou

**Affiliations:** ^1^State Key Laboratory of Virology, Hubei Province Key Laboratory of Allergy and Immunology, School of Basic Medical Sciences, Institute of Medical Virology, Wuhan University, Wuhan, China; ^2^School of Basic Medicine, Hubei University of Science and Technology, Xianning, China; ^3^Department of Microbiology and Immunology, Sealy Center for Vaccine Development and Institute for Human Infections and Immunity, University of Texas Medical Branch, Galveston, TX, United States; ^4^Department of Pathology and Laboratory Medicine, Temple University Lewis Katz School of Medicine, Philadelphia, PA, United States

**Keywords:** opioids, HIV, innate immunity, interferons, miRNAs

## Abstract

Opioid abuse alters the functions of immune cells in both *in vitro* and *in vivo* systems, including macrophages. Here, we investigated the effects of methadone, a widely used opioid receptor agonist for treatment of opiate addiction, on the expression of intracellular viral restriction factors and HIV replication in primary human macrophages. We showed that methadone enhanced the HIV infectivity in primary human macrophages. Mechanistically, methadone treatment of macrophages reduced the expression of interferons (IFN-β and IFN-λ2) and the IFN-stimulated anti-HIV genes (APOBEC3F/G and MxB). In addition, methadone-treated macrophages showed lower levels of several anti-HIV microRNAs (miRNA-28, miR-125b, miR-150, and miR-155) compared to untreated cells. Exogenous IFN-β treatment restored the methadone-induced reduction in the expression of the above genes. These effects of methadone on HIV and the antiviral factors were antagonized by pretreatment of cells with naltrexone. These findings provide additional evidence to support further studies on the role of opiates, including methadone, in the immunopathogenesis of HIV disease.

## Introduction

Injection drug use (IDU) is known as a major risk factor for spreading HIV infection and is a global public health concern (1). Methadone maintenance treatment (MMT) is an opioid substitution treatment for opiate addiction and IDU. Evidence has shown that MMT substantially decreases sharing of injecting devices, the frequency of drug use, drug crimes, and HIV transmission (2, 3). Since the mid-1990s, antiretroviral therapy (ART) has been widely applied to the treatment for HIV patients, which leads to extensive reductions in HIV-related morbidity and mortality (4, 5). However, high and sustained levels of medication adherence are required for the effectiveness treatment, which can be challenging for HIV patients (6, 7). Furthermore, drug users have lower medication adherence of ART and more rapid HIV disease progression compared to other HIV patients (8–10). Recent studies have found that ART obtains improved access and adherence to treatment when used in combination with MMT (11–13). On the whole, MMT not only decreases illicit opioid use and hence reduces HIV acquisition but is critical to improving the medication adherence to ART (14). Therefore, it is becoming more considerable to study the relationship between methadone and HIV.

As an opioid receptor agonist, methadone possesses pharmacological effects similar to opiates, which have a potential cofactor role in the immunopathogenesis of HIV disease (15–17). Accumulating evidence suggests that abuse of opiates (morphine and heroin) disturbs the function of the immune system, weakens the host's defense to HIV infection, and impairs the function of multiple organs (15–17). Several studies have reported that opioids enhance HIV infection through several mechanisms, including upregulation of HIV coreceptors (CCR5 and CXCR4) (18), inflammatory cytokines (19, 20), inhibition of IFNs, and IFN stimulating genes (21, 22). Importantly, *in vitro* and *in vivo* studies have shown that methadone may dysregulate the immune functions of mononuclear phagocytes (23), human T lymphocytes (24, 25), and NK cells (26). Although there was one report that methadone enhanced HIV replication in macrophages through upregulation of CCR5, a key coreceptor for HIV entry into target cells (23), it is still unclear whether methadone has the other mechanisms on host cell-mediated innate immunity against HIV infection. Therefore, we initiated this study to explore the impact of methadone on the intracellular immunity and HIV infection of primary human macrophages.

## Materials and Methods

### Blood Monocyte-Derived Macrophages

Human peripheral blood was obtained from healthy adult donors with no history of drug abuse. Written informed consent was signed by all the study participants. The Research Ethics Committee of School of Basic Medical Sciences of Wuhan University approved this project. Purification of monocytes was performed as described previously (27). Briefly, blood was layered over lymphocyte separation medium (Alere Technologies AS, Oslo, Norway) and centrifuged at 800 g for 30 min at room temperature. The gradient of peripheral blood mononuclear cells (PBMC) were obtained above the Ficoll layer, carefully aspirated, and then transferred to gelatin-coated flasks for cellular adherence. After 45 min incubation in 5% CO_2_ at 37°C, flasks were washed 8–10 times with DMEM to eliminate non-adherent cells. Cells were then exposed to 10 mM EDTA in DMEM containing 20% fetal calf serum. Freshly isolated monocytes were cultured in 48-well plates at a density of 2.5 × 10^5^ cells/well in Dulbecco's modified Eagle medium (DMEM) supplemented with 10% FBS, 2 mM glutamine, 100 U/mL streptomycin/penicillin and 1 μM GM-CSF. The medium was replaced at 2–3 days intervals, and monocyte-derived macrophages were obtained after 7 days culture. Macrophages were stained with fluorescence-conjugated anti-human CD14 antibody and analyzed for CD14 expression by flow cytometry. The purity of the macrophages was >95% according to flow cytometry analysis (Figure S1).

### Cytotoxicity Assay

The influence of methadone on cell viability was examined by 3-(4,5-dimethyl-2-thiazolyl)-2,5-diphenyl-2-H-tetrazolium bromide (MTT) assay. Macrophages were first seeded in 96-well plates and then treated with methadone at the indicated concentrations (0, 1, 10, 25, 50, 100, and 200 μM) for 24 h. MTT solution (20 μL, 5 mg/mL, Sigma) was added to the wells and re-incubated for 4 h at 37°C. Then medium was removed and 100 μL of dimethyl sulfoxide (Sigma) was added into each well. The optical density (OD) at 560 nm was measured by an ELISA reader. All assays were repeated three times.

### Methadone Treatment and HIV Infection

Methadone was kindly provided by Drug Rehabilitation Center of Wuhan Centers for Disease Control and Prevention. The macrophage-tropic R5 strain (Bal) was maintained in our laboratory and routinely propagated in primary human macrophages. Macrophages (2.5 × 10^5^ cells/well) were treated with different concentrations (0.01, 0.1, 1.0, 5.0, and 10.0 μM) of methadone for 24 h before infection with HIV. Naltrexone (1 μM, 3B Scientific Corporation, Wuhan, China), a μ-opioid receptor antagonist, was added to the macrophage cultures for 1 h prior to methadone treatment. After drug pre-treatment, the cells were infected with HIV (Bal strain, p24, 10 ng/10^6^ cells) for 2 h at 37°C and then washed with DMEM to remove the unabsorbed virus. Fresh medium containing methadone was added to cell cultures every 3 days. The cell culture supernatants were collected to assess HIV p24 production by using p24 ELISA kit (Zeptometrix Corp., Buffalo., NY., USA) at 2, 4, 8, and 12 days post infection (dpi). Cellular RNA and lysates were collected at the indicated time points for subsequent gene and protein detection, respectively.

### RNA/DNA Extraction and Real-Time RT-PCR

Total RNA from macrophages was extracted with TRIzol reagent (Invitrogen, Carlsbad, CA, USA) according to the manufacturer's instruction. Total RNA (1 μg) was subjected to reverse transcription using Moloney murine leukemia virus (M-MLV) reverse transcriptase (Promega, Madison, WI, USA) with random primers for 1 h at 42°C. The reaction was terminated by incubating the reaction mixture at 72°C for 10 min and then kept at 4°C. Total DNA from macrophages was extracted with Genomic DNA kit (CWBIO, Jiangsu, China) according to the manufacturer's instruction. The resulting cDNA or DNA was then used as a template for real-time PCR quantification with the iTaq Universal SYBR Green Supermix (Bio-Rad, Hercules, CA, USA) on a CFX Connect Real-Time PCR System (Bio-Rad). Primers sequences used in PCR reactions are shown in Table 1 and synthesized by Genecreate Biological Engineering Co (Wuhan, China). Gene expression was normalized to GAPDH and calculated using the 2^−ΔΔCt^ method. The U6 gene was used as an endogenous control for miRNAs detection.

**Table 1:** The primers for real-time RT-PCR.

**Target gene**	**primer**	**Nucleotide sequence**
GAPDH	F	5′-GGTGGTCTCCTCTGACTTCAACA-3′
	R	5′-GTTGCTGTAGCCAAATTCGTTGT-3′
GAG	F	5′ATAATCCACCTATCCCAGTAGGAGAA-3′
	R	5′-TTTGGTCCTTGTCTTATGTCCAGAAT-3′
MIP-1β	F	5′-CCAAACCAAAAGAAGCAAGC-3′
	R	5′-AGAAACAGTGACAGTGGACC-3′
IFN β	F	5′-GCACAACAGGTAGTAGGCGA-3′
	R	5′-GCCTCCCATTCAATTGCCAC-3′
IFN-λ2	F	5′-TTTAAGAGGGCCAAAGATGC-3′
	R	5′-TGGGCTGAGGCTGGATACAG-3′
APOBEC3G	F	5′-TCAGAGGACGGCATGAGACTTAC-3′
	R	5′-AGCAGGACCCAGGTGTCATTG-3′
APOBEC3F	F	5′-TTCGAGGCCAGGTGTATTCC-3′
	R	5′- GGCAGCTGGTTGCCACAGA-3′
MxB	F	5′-AGCAGTATCGAGGCAAGGAGC-3′
	R	5′-TGGCGAGACGTTTGCTGGTTTC-3′
miRNA-28	F	5′-ACACTCCAGCTGGGAAGGAGCTCACAGTCT-3′
	R	5′-TGGTGTCGTGGAGTCG-3′
miRNA-125b	F	5′-ACACTCCAGCTGGGTCCCTGAGACCCTAAC-3′
	R	5′-TGGTGTCGTGGAGTCG-3′
miRNA-150	F	5′-ACACTCCAGCTGGGTCTCCCAACCCTTGTA-3′
	R	5′-TGGTGTCGTGGAGTCG-3′
miRNA-155	F	5′-ACACTCCAGCTGGGTTAATGCTAATCGTGAT-3′
	R	5′-TGGTGTCGTGGAGTCG-3′
Strong stop	F	5′-GGCTAACTAGGGAACCCACTG-3′
	R	5′-CTGCTAGAGATTTTCCACACTGA-3′
Late RT	F	5′-TGTGTGCCCGTCTGTTGTGT-3′
	R	5′-GAGTCCTGCGTCGAGAGAGC-3′

### Western Blot and ELISA Analysis

Macrophages were washed with ice-cold PBS three times and lysed in RIPA with 1% protease inhibitor PMSF. Protein concentrations were determined by BCA protein assay. Proteins were resuspended in SDS-PAGE Sample Loading Buffer (Biosharp, Hefei, Anhui, China; BL502A) and heated for 5 min at 95°C. The equivalent amounts of protein (50 μg) for each sample were then resolved by 12% SDS-PAGE and transferred to PVDF membranes. The blots were subsequently immunoblotted with the following antibodies: anti-APOBEC3G (1:500; Santa Cruz Biotechnology, Santa Cruz, California, USA; sc-48820), anti-APOBEC3F (1:200; Santa Cruz; sc-46725), anti-MxB (1:1000; Santa Cruz; sc-271527), and anti-GAPDH (1:5000; Sungene biotech, Tianjin, China; KM9002). The blots were incubated with the HRP-Goat anti-Rabbit secondary antibody (1:5000; Cell Signaling Technology, Shanghai, China; 7074s) or HRP-Goat anti-mouse secondary antibody (1:5000; Cell Signaling Technology, Shanghai; 7076), respectively. The bound antibodies were detected using Electro-Chemi-Luminescence Substrate Kit (Wuhan Servicebio Technology Co, Wuhan, Hubei, China), and the signal intensities of protein bands were analyzed by ImageJ software (Dr. Wayne Basband, National Institutes of Health, Bethesda, MD, USA). Human MIP-1β, IFN-β, IFN-λ2 (4A Biotech, Beijing, China) and viral particles p24 (Zeptometrix Corp. Buffalo. NY. USA) in the supernatants were detected using ELISA kits according to the manufacturer's instruction.

### Statistical Analysis

Student's *t*-test was used to evaluate the significance of difference between groups, and multiple comparisons were performed by regression analysis and one-way analysis of variance. Statistical analyses were performed with Prism Software, and all data were presented as mean ± SD. Statistical significance was defined as *p* < 0.05.

## Result

### Methadone Enhances HIV Replication in Macrophages

Macrophages were treated with different concentrations of methadone (10–200 μM), and drug cytotoxicity was examined by MTT assay. Methadone treatment had little effect on viability of macrophages at concentrations lower than 10 μM and the median cytotoxic concentration (CC_50_) of methadone was 437.56 μM (Table 2). Thus, we utilized methadone at concentrations of 10 μM or lower throughout the following experiments.

**Table 2:** Cytotoxicity of methadone in macrophages.

	**Methadone (μM)**						**CC_**50**_ (μM)**
	**1**	**10**	**25**	**50**	**100**	**200**	
Cell viability (%)	99.62 ± 0.85	94.85 ± 2.26	88.69 ± 1.47	84.07 ± 1.51	79.50 ± 1.27	74.50 ± 0.86	437.56

**Figure 1:**
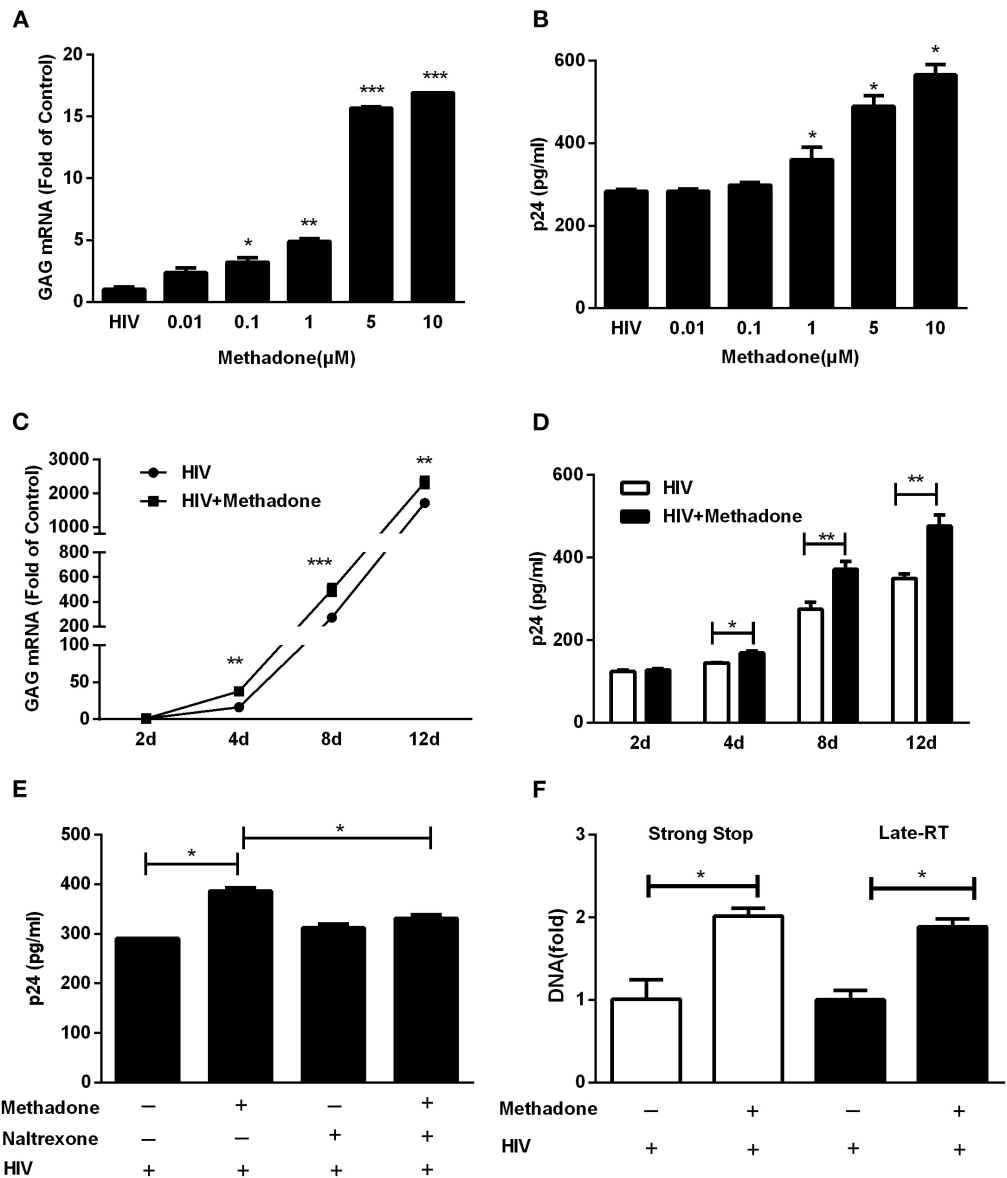
Methadone enhanced HIV infection of macrophages. Macrophages were treated with methadone at indicated concentrations for 24 h, and then infected with HIV Bal for 2 h. Total cellular RNA extracted from cells was collected for HIV GAG gene by real-time RT-PCR **(A)**, and culture supernatant collected at 8 dpi were analyzed by p24 ELISA **(B)**. Macrophages were treated with methadone (1 μM) for 24 h, and then infected with HIV Bal strain for 2 h. Cells and supernatants were collected at 2, 4, 8, and 12 dpi. Total cellular RNA extracted from cells was detected for HIV GAG gene by real-time RT-PCR **(C)**, and macrophages culture supernatants were analyzed by p24 ELISA **(D)**. Naltrexone (1 μM) was added to macrophages cultures for 1 h prior to methadone (1 μM) treatment. The cells were subsequently incubated with equal amounts of HIV Bal strain for 2 h, and fresh medium containing methadone (1 μM) with/without naltrexone (1 μM) was added to cell cultures. Macrophages culture supernatants were collected at 8 dpi and analyzed by ELISA **(E)**. **(F)** Macrophages were treated with methadone (1 μM) for 24 h, and then infected with HIV Bal for 2 h. Total cellular DNA extracted from cells was collected for HIV Strong Stop DNA and Late-RT DNA by real-time RT-PCR at 24 h post infection. The results are presented as means ± standard deviations obtained from three independent experiments (^***^p < 0.001; ^**^p < 0.01; ^*^p < 0.05).

To evaluate effect of methadone on HIV infection/replication, macrophages were incubated in the presence or absence of different concentrations of methadone (0.01, 0.1, 1.0, 5.0, and 10.0 μM) for 24 h and then challenged with HIV Bal strain for 2 h. As shown in Figure 1A, methadone significantly enhanced HIV GAG gene expression in macrophages in a dose-dependent manner at 8 dpi. In addition, methadone-treated macrophages showed higher levels of p24 protein compared to untreated cells (Figure 1B) at 8 dpi. Methadone (1 μM) significantly enhanced HIV GAG gene expression and p24 protein in macrophages in a time-dependent manner (Figures 1C,D). Pretreatment of macrophages with naltrexone, a μ-opioid receptor antagonist, blocked the methadone-induced upregulation of HIV p24 expression (Figure 1E) at 8 dpi, which indicated that this effect of methadone on HIV infection was acting through the opioid receptors.

Previous studies have demonstrated CCR5, the key coreceptor for HIV entry into the target cells, was elevated in methadone-treated macrophages compared to untreated cells (23). Here we also verified this phenomenon of methadone enhancement on CCR5 at both RNA and protein levels (Figure S2). Thus, we tested how methadone affected the early steps of HIV replication (virus entry and reverse transcription). Strong Stop DNA, the early product of reverse transcription synthesized shortly after viral entry, was used for the assessment of viral entry (28). Late-RT DNA was applied to monitor formation of reverse transcription products right after HIV entered the cell (29). As shown in Figure 1F, methadone enhanced HIV-1 Strong Stop DNA and reverse transcription DNA at 24 hpi, which implied that the critical step of HIV-1 infection elevated by methadone treatment occurred right after virus entry and initiation of reverse transcription.

### Methadone Reduces the Level of MIP-1β

**Figure 2:**
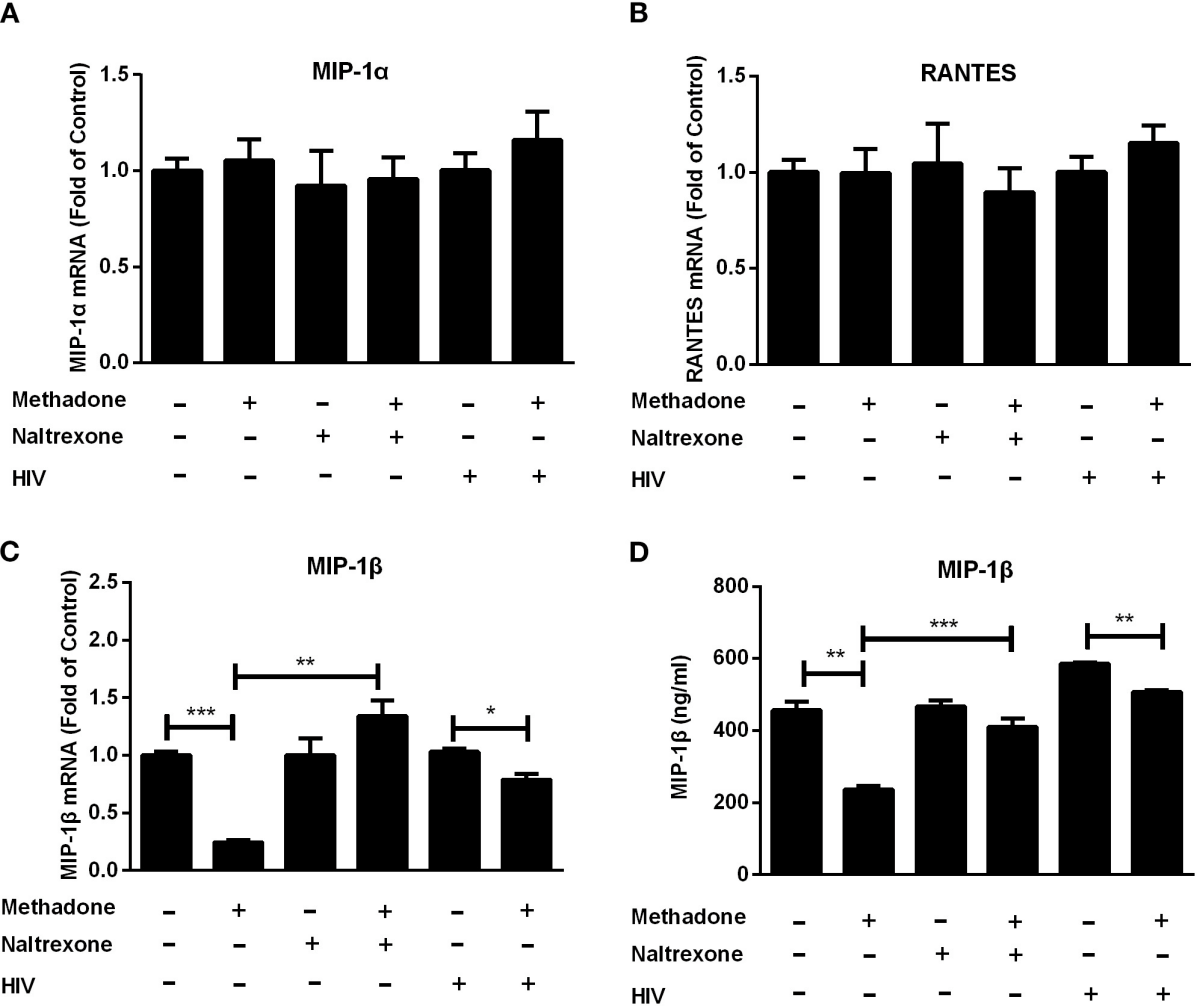
Methadone downregulated the expression of MIP-1β in macrophages. Naltrexone (1 μM) was added to macrophage cultures for 1 h prior to methadone (1 μM) treatment and macrophages and supernatants in groups without HIV were collected at 24 h post treatment. Macrophages in HIV groups were treated with or without methadone (1 μM) for 24 h and then infected with HIV Bal strain for 2 h. Cells and supernatants were collected at 8 dpi. Total cellular RNA was subjected to the real-time RT-PCR for MIP-1α **(A)**, RANTES **(B)**, and MIP-1β **(C)** mRNA, and GAPDH was used as a reference gene. **(D)** MIP-1β protein in the culture supernatant was analyzed by ELISA assay. The results are presented as means ± standard deviations obtained from three independent experiments (^***^p < 0.001; ^**^p < 0.01; ^*^p < 0.05).

Since CC chemokines (MIP-1α, MIP-1β, and RANTES) are the natural ligands for CCR5 (30), we investigated the effect of methadone on CC chemokines expression in macrophages. Macrophages were incubated with methadone for 24 h, and the intracellular gene expression of the CC chemokines (MIP-1α, MIP-1β, and RANTES) and protein level of the MIP-1β in the whole cell lysate were quantified by real-time RT-PCR and ELISA assay, respectively. As shown in Figure 2, although methadone had little effects on MIP-1α and RANTES (Figures 2A,B), methadone effectively inhibited the expression of MIP-1β in macrophages (75.5 ± 2.5% reduction in mRNA level; and 14.3 ± 2.8% reduction in protein level). Pretreatment of macrophages with naltrexone prior to methadone treatment eliminated the suppressive effect of methadone on MIP-1β expression at both mRNA and protein levels (the 4th column, Figures 2C,D), whereas the cell culture treated with naltrexone alone were not affected as to the CC chemokine expression (the 3rd column, Figures 2C,D). It is worth noting that methadone had the similar effect in HIV-infected macrophages regarding the inhibitory effect on MIP-1β expression at 8 dpi (the fifth and sixth columns, Figures 2C,D).

### Methadone Downregulates Interferon (IFN) Expression and Restriction Factors

**Figure 3:**
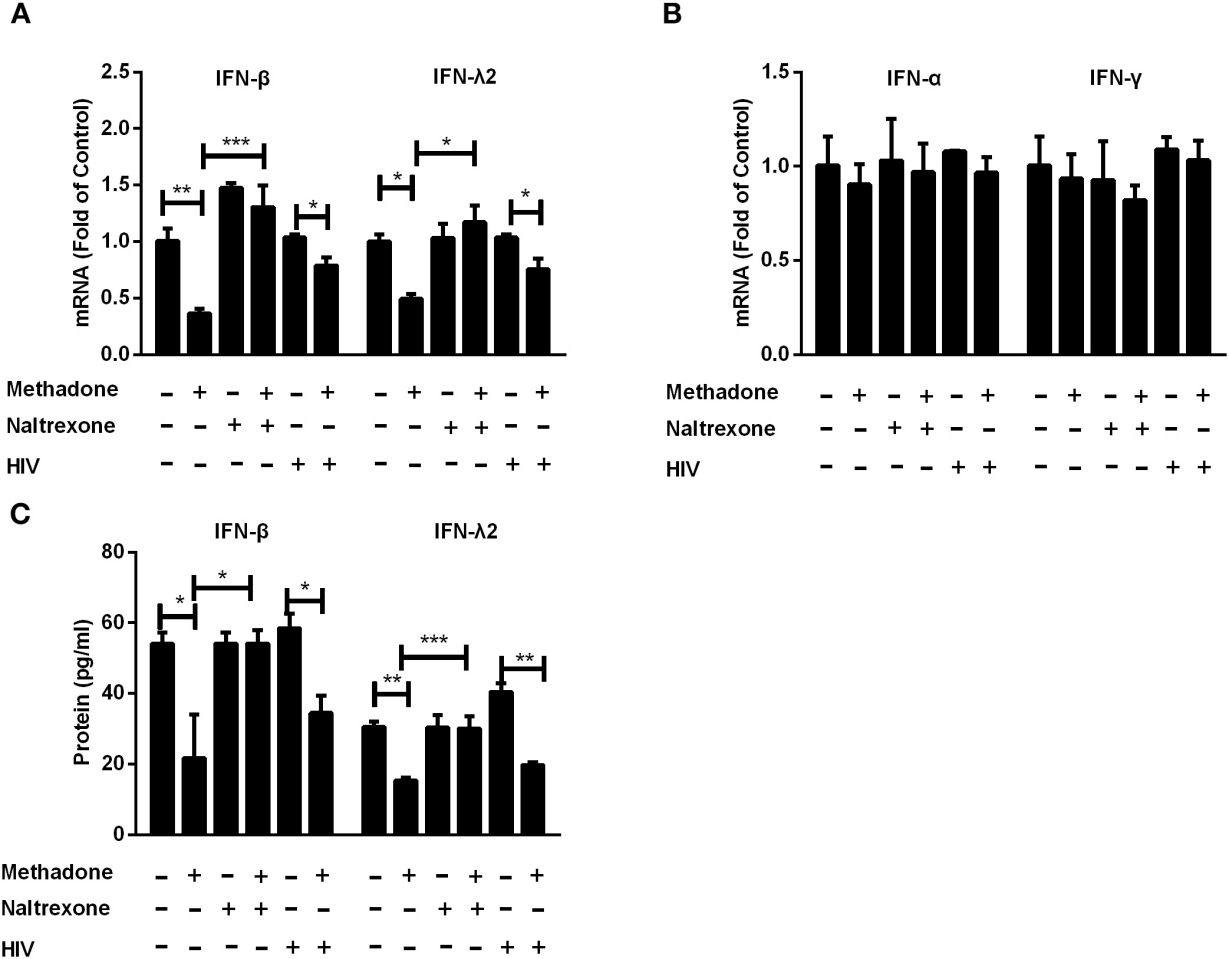
Methadone suppressed the expression of intracellular interferons in macrophages. Naltrexone (1 μM) was added to macrophages cultures for 1 h prior to methadone (1 μM) treatment. Macrophages and supernatants in groups without HIV were collected at 24 h post treatment. Macrophages in the HIV groups were treated with or without methadone (1 μM) for 24 h, and then infected with HIV Bal strain for 2 h. Cells and supernatants were collected at 8 dpi. Total cellular RNA was subjected to the real-time RT-PCR for IFN-β, IFN-λ2 **(A)**, IFN-α and IFN-γ **(B)**, and GAPDH was used as a reference gene. **(C)** Secretory IFN-β and IFN-λ2 in the culture supernatant were analyzed by ELISA assay. The results are presented as means ± standard deviations obtained from three independent experiments (^***^p < 0.001; ^**^p < 0.01; ^*^p < 0.05).

Since IFNs play an important role in host cell innate immunity against viral infections (31–34), we examined the effect of methadone on IFN expression in macrophages. Methadone treatment of macrophages significantly suppressed the expression of IFN-β (36.9 ± 0.7% reduction in mRNA level, Figure 3A; 53.8 ± 10% reduction in protein level, Figure 3C) and IFN-λ2 (50.8 ± 2.1% reduction in mRNA level, Figure 3A; 49.5 ± 5.3% reduction in protein level, Figure 3C), respectively. Pretreatment of macrophages with naltrexone dismissed the inhibitory effect of methadone on IFN-β and IFN-λ2 expression at both mRNA and protein levels, and naltrexone alone had little effect on IFN-β and IFN-λ2. However, methadone had little effect on the expression of IFN-α and IFN-γ in macrophages in our observation (Figure 3B). Moreover, we also found that methadone treatment of macrophages suppressed the expression of IFN-β and IFN-λ2 in the context of HIV infection (the fifth and sixth columns, Figures 3A,B).

**Figure 4:**
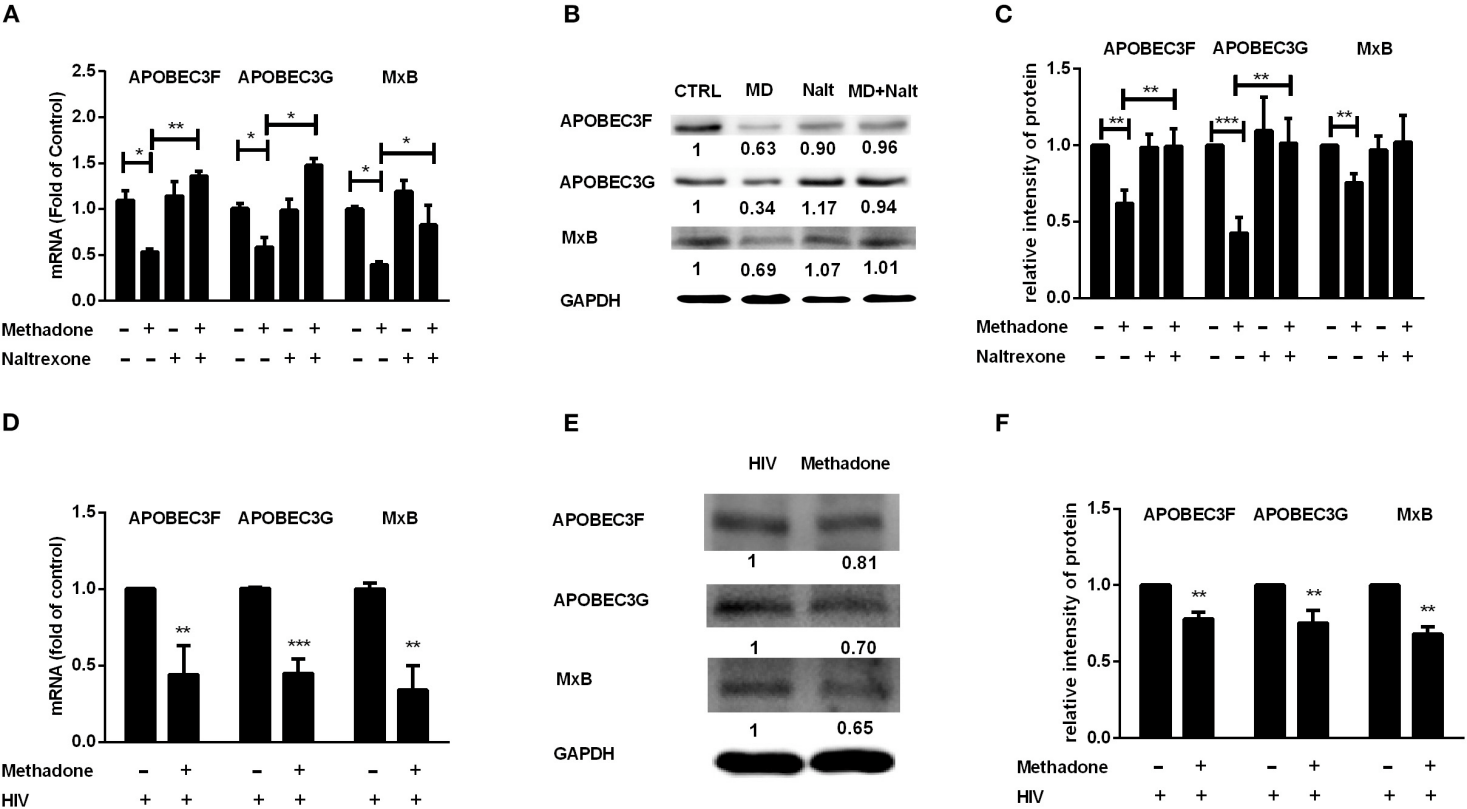
Methadone inhibited the expression of APOBEC3F, APOBEC3G, and MxB in macrophages. Naltrexone (Nalt, 1 μM) was added to macrophages cultures for 1 h prior to methadone (MD, 1 μM) treatment and macrophages in groups without HIV were collected at 24 h post treatment. Macrophages in HIV groups were treated with or without methadone (1 μM) for 24 h, and then infected with HIV Bal strain for 2 h, and cells were collected at 8 dpi. The expression of APOBEC3F, APOBEC3G, and MxB at the mRNA level **(A,D)** and protein level **(B,E)** were detected by real-time RT-PCR and Western blot, respectively. Intensities of protein bands of the Western blot were quantized after normalization with corresponding values of GAPDH expression **(C,F)**. The results are presented as means ± standard deviations obtained from three independent experiments (^***^p < 0.001; ^**^p < 0.01; ^*^p < 0.05).

Host restriction factors are an important and potent class of intracellular components to block to viral replication and initiate the innate immune response to viral infection. These host proteins are often induced by interferon signaling and antagonized by viral factors (35, 36). We further investigated the effect of methadone on the expression of APOBEC3F, APOBEC3G, and MxB in macrophages. We found macrophages treated with methadone showed significant reduction on the expressions of APOBEC3F (47.4 ± 15.6% reduction), APOBEC3G (40 ± 21.5% reduction), and MxB (60.7 ± 6.5% reduction) compared to untreated cells (Figure 4A). Western blot analysis further confirmed the methadone action on the protein production of APOBEC3G, APOBEC3F, and MxB, with 32.3 ± 3.8%, 43.9 ± 11%, and 61.8 ± 20% of downregulation, respectively (Figure 4B). The inhibitory effects of methadone can be blocked by pretreatment of macrophages with naltrexone (Figure 4). It is important to note that during HIV infection, methadone treatment of macrophages reduced the expression of APOBEC3G, APOBEC3F, and MxB in both mRNA and protein levels (Figures 4D–F).

### Methadone Inhibits HIV Restriction miRNAs

**Figure 5:**
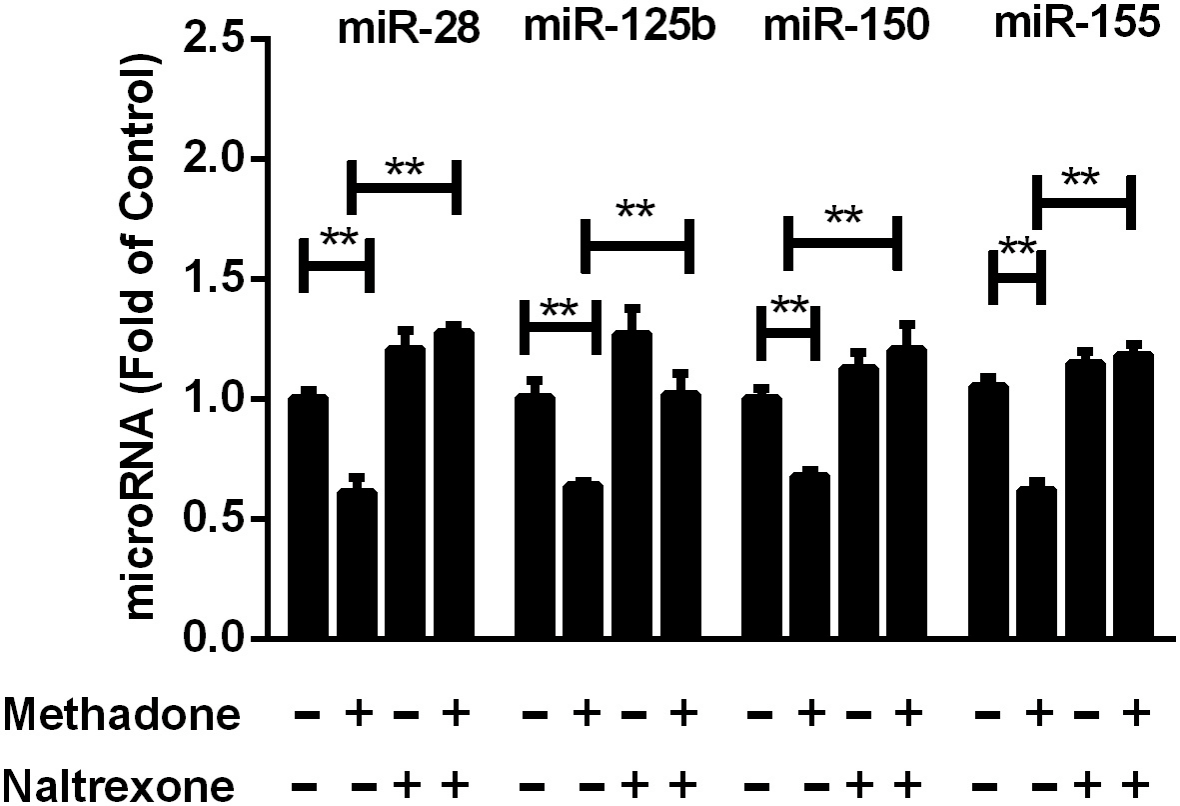
Methadone decreased the expression of anti-HIV microRNAs in macrophages. Naltrexone (1 μM) was added to macrophages cultures for 1 h prior to methadone (1 μM) treatment, and macrophages were collected at 24 h post treatment. Total RNA extracted from the cells was subjected to the real-time RT-PCR for the expression of anti-HIV miRNAs (miR-28, miR-125b, miR-150, and miR-155). The results are presented as means ± standard deviations obtained from three independent experiments (^**^p < 0.01).

Previous studies have shown that certain cellular miRNAs contribute to restriction of HIV replication in ${\text{CD}}_{4}^{+}$ T lymphocytes (37), monocytes and macrophages (38). Thus, we assessed the effect of methadone on the expression of the HIV restriction miRNAs (miRNA-28, miRNA-125b, miRNA-150, and miRNA-155) in macrophages. We demonstrated that methadone reduced the expression of these miRNAs compared to untreated cells (Figure 5). Pretreatment of macrophages with naltrexone diminished the suppressive effect of methadone on the HIV restriction miRNAs, whereas naltrexone alone had little effect on these miRNAs (Figure 5).

### IFN-β Addition Restores the Decreased Gene Expression Caused by Methadone

**Figure 6:**
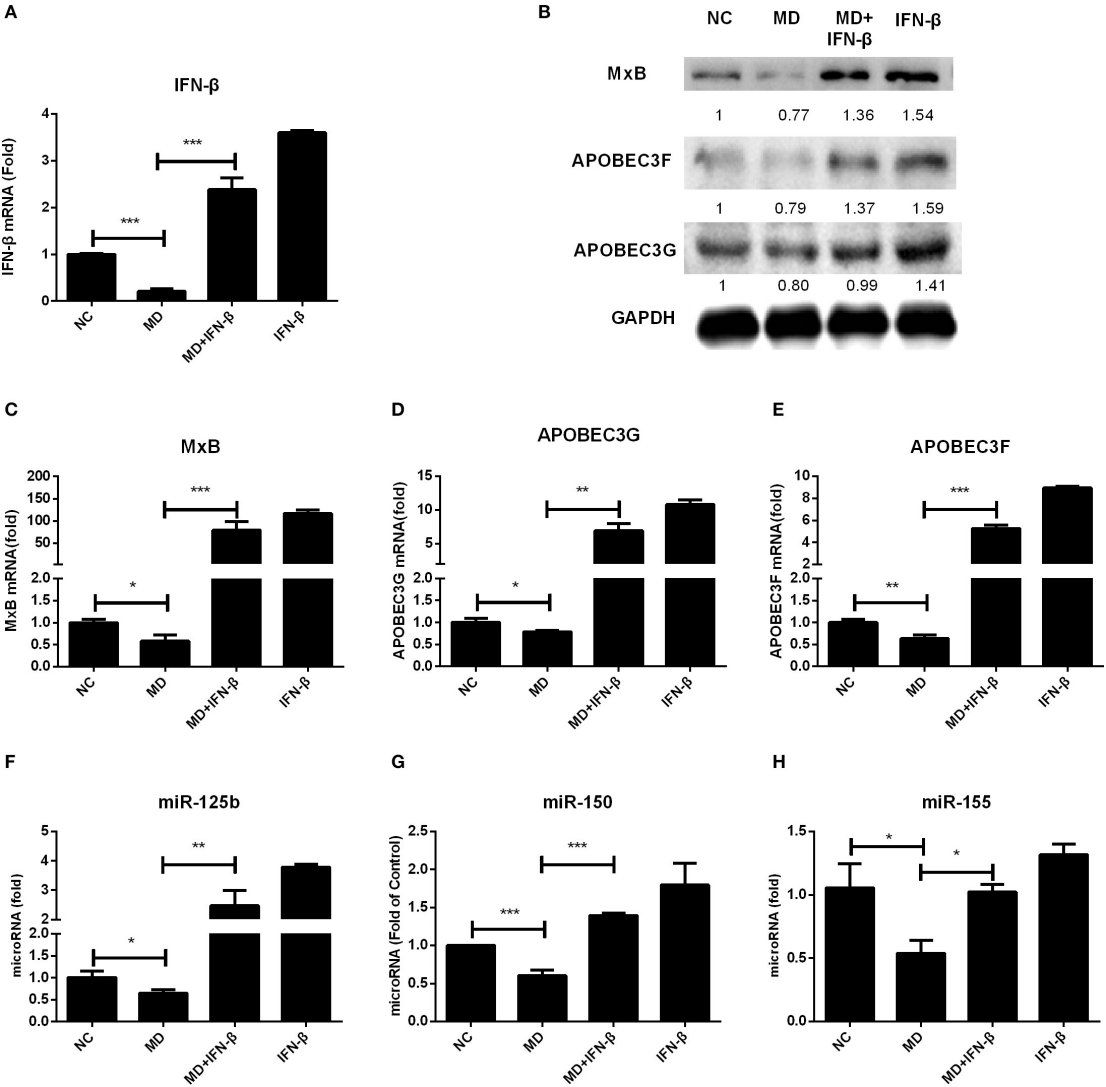
IFN-β addition restores the decreased gene expression caused by methadone. Macrophages were treated with methadone (1 μM), methadone combining with IFN-β (100 U/ml) or IFN-β (100 U/ml) for 24 h. Total cellular RNA extracted from cells was collected for IFN-β, APOBEC3G, APOBEC3F, MxB, and miRNAs (miRNA-125b, miRNA-150, and miRNA-155) gene by real-time RT-PCR **(A, C–H)** and the protein collected at 24 h was analyzed by Western blot **(B)**. The results are presented as means ± standard deviations obtained from three independent experiments (^***^p < 0.001; ^**^p < 0.01; ^*^p < 0.05).

We already revealed the inhibitory effect of methadone in IFN production as described above, which raises an interesting question whether this IFN downregulation is directly linked to host restriction factors and miRNAs modulation. We utilized exogenous IFN-β treatment on methadone-treated cells and found that IFN-β restored the decreased gene expression of IFN-β caused by methadone (Figure 6A). Consequently, reduced ISGs due to methadone treatment, including APOBEC3G, APOBEC3F, and MxB, could be recovered by IFN-β addition at the mRNA (Figures 6C–E) and protein level (Figure 6B). A pack of HIV restriction miRNAs (miR-125b, miR-150, and miR-155) also reached elevated levels after IFN-β treatment (Figures 6F–H). Therefore, we concluded that IFN-β addition reverted the inhibitory effect of methadone on restriction factors (APOBEC3G, APOBEC3F, and MxB) and miRNAs (miR-125b, miR-150, and miR-155).

## Discussion

Methadone maintenance treatment reduces or eliminates craving for opioids, prevents opioid withdrawal symptoms, and blocks the euphoric effects of additional opioids. Therefore, methadone can widely be applied as an effective treatment for drug abusers (39–42). Furthermore, opioid abusers receiving MMT have better adherence to ART and lower likelihood of HIV infection associated with drug injection compared to non-MMT treated group (11–13, 43). The increasing reliance on MMT to control the HIV epidemic in IDUs calls for urgent clarification of the remaining uncertainties regarding methadone and immune function. Previous research indicated that illicit drugs (methamphetamine, morphine, and heroin), even alcohol, influenced HIV entry, activated the transcription of HIV LTR, and disordered innate immunity, thereby promoting HIV replication in human immune cells (44–51). However, not much is known about whether methadone can enhance virus infection like other drugs by regulating immune system in macrophages, one of the primary targets and reservoir of the HIV. In this study, our data supports the hypothesis that methadone augments HIV infection through the dysregulation of antiviral factors in macrophages.

In our experiments, we found that methadone facilitated HIV replication in macrophages (Figure 1) and enhanced the synthesis of HIV-1 Strong Stop DNA and reverse transcription DNA at 24 hpi, which signified that the key steps for HIV infection increased by methadone treatment occurred after the preliminary stage of virus entry. Studies have documented that morphine could enhance HIV replication in human immune cells by the modulation of beta-chemokines and CCR5 receptor (20, 50). In addition, Li et al. showed that, similar to morphine, methadone facilitated HIV activation and replication through the upregulation of CCR5 (23). Consistent with previous study, we also confirmed methadone significantly upregulated the expression of CCR5, which was abrogated by naltrexone treatment in macrophages (Figure S2). In the present study, we found that methadone treatment of macrophages suppressed the expression of MIP-1β (Figure 2), which is supported by other reports that how that morphine (50) and cocaine (52) inhibited β-chemokines expression in MDMs and PBMCs. It is well-known that the binding of HIV gp120 to both CD4 and a co-receptor is necessary for the entry of most HIV-1 strains. Additionally, β-chemokines, the natural ligands for co-receptor CCR5, interfere with HIV infection by competing for the CCR5 receptor in macrophages (53). Combined with the enhancement on the synthesis of Strong Stop DNA and reverse transcription DNA by methadone (Figure 1F), we assume that the upregulation of CCR5 and downregulation of MIP-1β production by methadone treatment may be directly responsible for the HIV enhancement by methadone in the early stage of HIV infectivity in macrophages.

Type I IFNs have been well-known to induce the expression of hundreds of IFN-stimulated genes (ISGs), including a variety of antiviral restriction factors as part of the innate immune response (54). We subsequently examined the effect of methadone on the expression of IFNs and restriction factors, which may contribute to understanding the potential mechanism responsible for the action of methadone. In our study, we found that methadone treatment of macrophages suppressed the expression of IFN-β and IFN-λ2 (Figure 3). This finding supports the earlier observation that morphine or heroin could significantly inhibit the expression of endogenous IFNs in macrophages (49, 55). APOBEC (apolipoprotein B mRNA-editing enzymecatalytic polypeptide-like protein) family members inhibit retroviruses replication by deamination to convert cytidine(C) to uridine (U) of viral DNA (56), among which APOBEC3F and APOBEC3G can restrict HIV replication in both CD4+ T cells and macrophages (57, 58). MxB directly binds the HIV capsid to interfere with virus nuclear entry/post-nuclear and prevents the uncoating process (59). In our finding, methadone may enhance HIV infection through inhibiting restriction factors (APOBEC3G/F and MxB) production and impairing intracellular innate antiviral mechanism in macrophages (Figure 4). These findings support the earlier report showing that morphine treatment of human macrophages significantly inhibited the expression of APOBEC3G/F (55). In addition to inducing the expression of restriction factors, IFN can also act as the regulator of some miRNAs to elicit broad anti-viral effects (60). MiRNA-28, miRNA-125b, and miRNA-150, which are known to target 3′UTR of HIV transcripts, induced HIV latency, and inhibited HIV-1 replication (37, 38). MiRNA-155 inhibits the entry and viral integration of HIV by reducing several cellular factors required for viral replication (61–63). We discovered that methadone reduced the expression of these miRNAs (miRNA-28, miRNA-125b, miRNA-150, and miRNA-155) compared to untreated cells. Collectively, we demonstrated that methadone inhibited the expression of IFN-β and IFN-λ2 in primary macrophages with/without HIV infection, which further regulated the subsequent restriction factors (APOBEC3F, APOBEC3G, and MxB) and other anti-HIV miRNAs (miR-28, miR-125b, miR-150, and miR-155).

To verify that IFN downmodulation is directly linked to host restriction factors and miRNAs modulation, we further confirmed that exogenous IFN-β treatment of macrophages significantly induced APOBEC3G, APOBEC3F, and MxB expression (Figure S3). Similarly, exogenous IFN-β also upregulated the expression of some miRNAs (miRNA-125b, miRNA-150, and miRNA-155) in macrophages (Figure S3). What is more, IFN-β addition to methadone-treated cells restored the decreased gene expression (APOBEC3G, APOBEC3F, MxB, miR-125b, miR-150, and miR-155) caused by methadone (Figure 6). Therefore, we conclude that it is likely that the downregulation of restriction factors and miRNAs caused by methadone is due to the negative effect on IFN-β.

The interactions between opioid and immune system are complex and their different immunological outcomes depend heavily on the molecular structure of the opioids, the profiles of receptor binding, and the modulation of immune effector cells and molecules. Opioids predominantly exert effects via the μ opioid receptors (MOR) pathway. In our experiments, this methadone action was also mediated by MOR because the naltrexone, a MOR antagonist, blocked the effect of methadone on HIV infection and gene expression (MIP-1β, IFN-β, restriction factors, and miRNAs) of macrophages. In addition, dopamine D1 receptor (D1R) has been implicated in mediating the dopaminergic neurotoxicity and IFN-α expression triggered by methamphetamine, suggesting that opioids may regulate immune system via D1R (44, 64). Opioids also have differential effects on immune cells, including neutrophils, monocytes, NK cells, and T cells (23–26). Several studies have reported that opioids accommodate immune function through several mechanisms including upregulation of HIV coreceptor (CCR5 and CXCR4) (18), regulation of inflammatory cytokines (19, 20), inhibition of IFNs and IFN stimulating genes (21, 22), motivation of the Toll-like receptor 4 (TLR4) signaling pathway (65, 66), and modulation of HLA-DR in dendritic cells (67). As an opioid agonist, methadone may have the direct and indirect immunoregulatory effects similar to other opioids, thus affect susceptibility to HIV infection. Our research has enriched and improved the understanding of interaction between methadone and immune system during HIV infection.

Taken together, our work demonstrates that methadone potentiates HIV replication in primary macrophages in both dose- and time-dependent manner. One possible explanation is that methadone enhances the expression of coreceptor CCR5 and inhibits the production of the specific ligand MIP-1β, which directly results in the increasing entry of HIV to host cell as further evidenced by the accumulative amount of Strong Stop DNA and reverse transcription DNA. Besides, another potential mechanism is the inhibition on the IFN-β and IFN-λ2, which further regulates the subsequent restriction factors (APOBEC3F, APOBEC3G, and MxB) and other anti-HIV miRNAs (miR-28, miR-125b, miR-150, and miR-155). These modulating effects of methadone are largely blocked by naltrexone, suggesting that methadone action is mediated through μ-opioid receptor. These observations provide a novel mechanism for methadone-mediated HIV enhancement in primary human macrophages. Given the limitation of our *in vivo* studies, future *ex vivo* and *in vivo* studies are necessary and critical in order to determine the clinical impact of methadone on the immunopathogenesis of HIV disease.

## Data Availability Statement

All datasets generated for this study are included in the article/Supplementary Material.

## Ethics Statement

Written informed consent was signed by all the study participants. The Research Ethics Committee of School of Basic Medical Sciences of Wuhan University approved this project.

## Author Contributions

D-DW, H-RX, and WH conceived and designed the experiment. M-RW, D-DW, FL, Y-JW, XiW, and NZ performed the experiments. YF, XuW, and WH analyzed the data. H-TH and WH contributed reagents, materials, analysis tools. M-RW, D-DW, H-RX, and WH wrote the paper. All authors contributed to the article and approved the submitted version.

## Conflict of Interest

The authors declare that the research was conducted in the absence of any commercial or financial relationships that could be construed as a potential conflict of interest.

## Abbreviations

IFN: interferon
MIP-1α: macrophage inflammatory protein 1 alpha
MIP-1β: macrophage inflammatory protein 1 beta
RANTES: regulated on activation normal T cell expressed and secreted
APOBEC3G/3F: apolipoprotein B mRNA-editing enzyme-catalytic polypeptide-like 3G/3F
GAPDH: glyceraldehyde phosphate dehydrogenase
MxB: myxovirus resistance protein 2
ISG: interferon-stimulated gene.

## References

[1] Karon JM Fleming PL Steketee RW De Cock KM. HIV in the United States at the turn of the century: an epidemic in transition. Am J Public Health. (2001) 91:1060–8. 10.2105/AJPH.91.7.1060 11441732 PMC1446722

[2] Wodak A McLeod L. The role of harm reduction in controlling HIV among injecting drug users. AIDS. (2008) 22:S81–9. 10.1097/01.aids.0000327439.20914.33 18641473 PMC3329723

[3] Zhang L Zou X Xu Y Medland N Deng L Liu Y. The decade-long chinese methadone maintenance therapy yields large population and economic benefits for drug users in reducing harm, HIV and HCV disease burden. Front Public Health. (2019) 7:327. 10.3389/fpubh.2019.00327 31781529 PMC6861367

[4] Egger M May M Chene G Phillips AN Ledergerber B Dabis F. Prognosis of HIV-1-infected patients starting highly active antiretroviral therapy: a collaborative analysis of prospective studies. Lancet. (2002) 360:119–29. 10.1016/s0140-6736(02)09411-4 12126821

[5] Hogg RS Yip B Chan KJ Wood E Craib KJ O'Shaughnessy MV. Rates of disease progression by baseline CD4 cell count and viral load after initiating triple-drug therapy. JAMA. (2001) 286:2568–77. 10.1001/jama.286.20.2568 11722271

[6] Mannheimer SB Matts J Telzak E Chesney M Child C Wu AW. Terry Beirn Community Programs for clinical research on: quality of life in HIV-infected individuals receiving antiretroviral therapy is related to adherence. AIDS Care. (2005) 17:10–22. 10.1080/09540120412331305098 15832830

[7] Altice FL Mostashari F Friedland GH. Trust and the acceptance of and adherence to antiretroviral therapy. J Acquir Immune Defic Syndr. (2001) 28:47–58. 10.1097/00042560-200109010-00008 11579277

[8] Lucas GM Griswold M Gebo KA Keruly J Chaisson RE Moore DR. Illicit drug use and HIV-1 disease progression: a longitudinal study in the era of highly active antiretroviral therapy. Am J Epidemiol. (2006) 163:412–20. 10.1093/aje/kwj059 16394200

[9] Lucas GM Cheever LW Chaisson RE Moore RD. Detrimental effects of continued illicit drug use on the treatment of HIV-1 infection. J Acquir Immune Defic Syndr. (2001) 27:251–9. 10.1097/00126334-200107010-00006 11464144

[10] Wood E Montaner JSG Tyndall MW Schechter MT O'Shaughnessy MV Hogg RS. Prevalence and correlates of untreated human immunodeficiency virus type 1 infection among persons who have died in the era of modern antiretroviral therapy. J Infect Dis. (2003) 188:1164–70. 10.1086/378703 14551887

[11] Uhlmann S Milloy MJ Kerr T Zhang R Guillemi S Marsh D. Methadone maintenance therapy promotes initiation of antiretroviral therapy among injection drug users. Addiction. (2010) 105:907–13. 10.1111/j.1360-0443.2010.02905.x 20331553 PMC2857602

[12] Roux P Carrieri MP Villes V Dellamonica P Poizot-Martin I Ravaux I. The impact of methadone or buprenorphine treatment and ongoing injection on highly active antiretroviral therapy (HAART) adherence: evidence from the MANIF2000 cohort study. Addiction. (2008) 103:1828–36. 10.1111/j.1360-0443.2008.02323.x 18778390

[13] Palepu A Tyndall MW Joy R Kerr T Wood E Press N. Antiretroviral adherence and HIV treatment outcomes among HIV/HCV co-infected injection drug users: the role of methadone maintenance therapy. Drug Alcohol Depend. (2006) 84:188–94. 10.1016/j.drugalcdep.2006.02.003 16542797

[14] Donny EC Walsh SL Bigelow GE Eissenberg T Stitzer LM. High-dose methadone produces superior opioid blockade and comparable withdrawal suppression to lower doses in opioid-dependent humans. Psychopharmacology. (2002) 161:202–12. 10.1007/s00213-002-1027-0 11981600

[15] Donahoe RM Vlahov D. Opiates as potential cofactors in progression of HIV-1 infections to AIDS. J Neuroimmunol. (1998) 83:77–87. 10.1016/S0165-5728(97)00224-5 9610676

[16] Risdahl JM Khanna KV Peterson PK Molitor WT. Opiates and infection. J Neuroimmunol. (1998) 83:4–18. 10.1016/S0165-5728(97)00216-6 9610668

[17] Kapadia F Vlahov D Donahoe RM Friedland G. The role of substance abuse in HIV disease progression: reconciling differences from laboratory and epidemiologic investigations. Clin Infect Dis. (2005) 41:1027–34. 10.1086/433175 16142670

[18] Steele AD Henderson EE Rogers JT. μ-opioid modulation of HIV-1 coreceptor expressionand HIV-1 replication. Virology. (2003) 309:99–107. 10.1016/S0042-6822(03)00015-1 12726730

[19] Alicea C Belkowski S Eisenstein TK Adler MW Rogers JT. Inhibition of primary murine macrophage cytokine production *in vitro* following treatment with the kappa-opioid agonist U50,488H. J Neuroimmunol. (1996) 64:83–90. 10.1016/0165-5728(95)00159-X 8598393

[20] Mahajan SD Schwartz SA Shanahan TC Chawda RP Nair NMP. Morphine regulates gene expression of - and -chemokines and their receptors on Astroglial cells via the opioid receptor. J Immunol. (2002) 169:3589–99. 10.4049/jimmunol.169.7.3589 12244149

[21] Li Y Ye L Peng JS Wang CQ Luo GX Zhang T. Morphine inhibits intrahepatic interferon- alpha expression and enhances complete hepatitis C virus replication. J Infect Dis. (2007) 196:719–30. 10.1086/520093 17674315

[22] Wan Q Wang X Wang YJ Song L Wang SH Ho ZW. Morphine suppresses intracellular interferon-alpha expression in neuronal cells. J Neuroimmunol. (2008) 199:1–9. 10.1016/j.jneuroim.2008.04.026 18562017 PMC2535790

[23] Li Y Wang X Tian S Guo CJ Douglas SD Ho ZW. Methadone enhances human immunodeficiency virus infection of human immune cells. J Infect Dis. (2002) 185:118–22. 10.1086/338011 11756991 PMC4009627

[24] Borner C Lanciotti S Koch T Hollt V Kraus J. mu opioid receptor agonist-selective regulation of interleukin-4 in T lymphocytes. J Neuroimmunol. (2013) 263:35–42. 10.1016/j.jneuroim.2013.07.012 23965172

[25] Kafami L Etesami I Felfeli M Enayati N Ghiaghi R Aminian A. Methadone diminishes neuroinflammation and disease severity in EAE through modulating T cell function. J Neuroimmunol. (2013) 255:39–44. 10.1016/j.jneuroim.2012.10.015 23177720

[26] Klimas NG Blaney NT Morgan RO Chitwood D Milles K Lee H. Immune function and anti-HTLV-I/II status in anti-HIV-1-negative intravenous drug users receiving methadone. Am J Med. (1991) 90:163–70. 10.1016/0002-9343(91)80155-F 1671730

[27] Hou W Ye L Ho ZW. CD56+ T cells inhibit HIV-1 infection of macrophages. J Leukoc Biol. (2012) 92:343–51. 10.1189/jlb.0312146 22591692 PMC4050632

[28] Xu XQ Guo L Wang X Liu Y Liu H Zhou RH. Human cervical epithelial cells release antiviral factors and inhibit HIV replication in macrophages. J Innate Immun. (2019) 11:29–40. 10.1159/000490586 30032138 PMC6338329

[29] Butler SL Hansen MS Bushman FD. A quantitative assay for HIV DNA integration *in vivo*. Nat Med. (2001) 7:631–4. 10.1038/87979 11329067

[30] Jin J Colin P Staropoli I Lima-Fernandes E Ferret C Demir A. Targeting spare CC chemokine receptor 5 (CCR5) as a principle to inhibit HIV-1 entry. J Biol Chem. (2014) 289:19042–52. 10.1074/jbc.M114.559831 24855645 PMC4081942

[31] Shirazi Y Pitha MP. Alpha interferon inhibits early stages of the human immunodeficiency virus type 1 replication cycle. J Virol. (1992) 66:1321–8. 10.1128/JVI.66.3.1321-1328.1992 1738192 PMC240853

[32] Taniguchi T Takaoka A. The interferon-alpha/beta system in antiviral responses: a multimodal machinery of gene regulation by the IRF family of transcription factors. Curr Opin Immunol. (2002) 14:111–6. 10.1016/S0952-7915(01)00305-3 11790540

[33] Gessani S Puddu P Varano B Borghi P Conti L Fantuzzi L. Role of endogenous interferon-beta in the restriction of HIV replication in human monocyte/macrophages. J Leukoc Biol. (1994) 56:358–61. 10.1002/jlb.56.3.358 8083608

[34] Sen GC Viruses and interferons. Annu Rev Microbiol. (2001) 55:255–81. 10.1146/annurev.micro.55.1.255 11544356

[35] Duggal NK Emerman M. Evolutionary conflicts between viruses and restriction factors shape immunity. Nat Rev Immunol. (2012) 12:687–95. 10.1038/nri3295 22976433 PMC3690816

[36] Hotter D Kirchhoff F. Interferons and beyond: Induction of antiretroviral restriction factors. J Leukoc Biol. (2018) 103:465–77. 10.1002/JLB.3MR0717-307R 29345347

[37] Huang J Wang F Argyris E Chen K Liang Z Tian H. Cellular microRNAs contribute to HIV-1 latency in resting primary CD4+ T lymphocytes. Nat Med. (2007) 13:1241–7. 10.1038/nm1639 17906637

[38] Wang X Ye L Hou W Zhou Y Wang YJ Metzger DS. Cellular microRNA expression correlates with susceptibility of monocytes/macrophages to HIV-1 infection. Blood. (2009) 113:671–4. 10.1182/blood-2008-09-175000 19015395 PMC2628373

[39] Bart G. Maintenance medication for opiate addiction: the foundation of recovery. J Addict Dis. (2012) 31:207–25. 10.1080/10550887.2012.694598 22873183 PMC3411273

[40] Fullerton CA Kim M Thomas CP Lyman DR Montejano LB Dougherty RH Medication-assisted treatment with methadone: assessing the evidence. Psychiatr Serv. (2014) 65:146–57. 10.1176/appi.ps.201300235 24248468

[41] Sees KL Delucchi KL Masson C Rosen A Clark HW Robillard H. Methadone maintenance vs 180-day psychosocially enriched detoxification for treatment of opioid dependence: a randomized controlled trial. JAMA. (2000) 283:1303–10. 10.1001/jama.283.10.1303 10714729

[42] Kreek MJ LaForge KS Butelman E. Pharmacotherapy of addictions. Nat Rev Drug Discov. (2002) 1:710–26. 10.1038/nrd897 12209151

[43] Mattick RP Breen C Kimber J Davoli M Methadone maintenance therapy versus no opioid replacement therapy for opioid dependence. Cochrane Database Syst Rev. (2009) 8:CD002209 10.1002/14651858.CD002209.pub2 12519570

[44] Liang H Wang X Chen H Song L Ye L Wang SH. Methamphetamine enhances HIV infection of macrophages. Am J Pathol. (2008) 172:1617–24. 10.2353/ajpath.2008.070971 18458095 PMC2408421

[45] Nair MP Saiyed MZ. Effect of methamphetamine on expression of HIV coreceptors and CC-chemokines by dendritic cells. Life Sci. (2011) 88:987–94. 10.1016/j.lfs.2010.09.019 20932494 PMC3044785

[46] Marcondes MC Flynn C Watry DD Zandonatti M Fox SH. Methamphetamine increases brain viral load and activates natural killer cells in simian immunodeficiency virus-infected monkeys. Am J Pathol. (2010) 177:355–61. 10.2353/ajpath.2010.090953 20489154 PMC2893678

[47] Toussi SS Joseph A Zheng JH Dutta M Santambrogio L Goldstein H. Short communication: Methamphetamine treatment increases *in vitro* and *in vivo* HIV replication. AIDS Res Hum Retroviruses. (2009) 25:1117–21. 10.1089/aid.2008.0282 19895343 PMC2828189

[48] Wang X Douglas SD Metzger DS Guo CJ Li Y O'Brien CP. Alcohol potentiates HIV-1 infection of human blood mononuclear phagocytes. Alcohol Clin Exp Res. (2002) 26:1880–6. 10.1111/j.1530-0277.2002.tb02496.x 12500113 PMC4015111

[49] Wang X Ma TC Li JL Zhou Y Geller EB Adler MW. Heroin inhibits HIV-restriction miRNAs and enhances HIV infection of macrophages. Front Microbiol. (2015) 6:1230. 10.3389/fmicb.2015.01230 26583016 PMC4632020

[50] Guo CJ Li Y Tian S Wang X Douglas SD Ho ZW. Morphine enhances HIV infection of human blood mononuclear phagocytes through modulation of beta-chemokines and CCR5 receptor. J Investig Med. (2002) 50:435–42. 10.1136/jim-50-06-03 12425430 PMC4037869

[51] Wang X Liu J Zhou L Ho ZW. Morphine withdrawal enhances HIV infection of macrophages. Front Immunol. (2019) 10:2601. 10.3389/fimmu.2019.02601 31803178 PMC6872497

[52] Nair MP Chadha KC Hewitt RG Mahajan S Sweet A Schwartz AS. Cocaine differentially modulates chemokine production by mononuclear cells from normal donors and human immunodeficiency virus type 1-infected patients. Clin Diagn Lab Immunol. (2000) 7:96–100. 10.1128/CDLI.7.1.96-100.2000 10618285 PMC95830

[53] Stantchev T Broder C. Human immunodeficiency virus type-1 and chemokine beyond competition for common cellular receptors. Cytokine Growth Factor Rev. (2001) 12:219–43. 10.1016/S1359-6101(00)00033-2 11325604

[54] Schoggins JW Rice MC. Interferon-stimulated genes and their antiviral effector functions. Curr Opin Virol. (2011) 1:519–25. 10.1016/j.coviro.2011.10.008 22328912 PMC3274382

[55] Wang Y Wang X Ye L Li J Song L Fulambarkar N. Morphine suppresses IFN signaling pathway and enhances AIDS virus infection. PLoS ONE. (2012) 7:e31167. 10.1371/journal.pone.0031167 22359571 PMC3281044

[56] Holmes RK Malim MH Bishop NK. APOBEC-mediated viral restriction: not simply editing? Trends Biochem Sci. (2007) 32:118–28. 10.1016/j.tibs.2007.01.004 17303427

[57] Mangeat B Turelli P Caron G Friedli M Perrin L Trono D. Broad antiretroviral defence by human APOBEC3G through lethal editing of nascent reverse transcripts. Nature. (2003) 424:99–103. 10.1038/nature01709 12808466

[58] Chiu YL Soros VB Kreisberg JF Stopak K Yonemoto W Greene CW. Cellular APOBEC3G restricts HIV-1 infection in resting CD4+ T cells. Nature. (2005) 435:108–14. 10.1038/nature03493 15829920

[59] Buffone C Schulte B Opp S Diaz-Griffero F Contribution of MxB oligomerization to HIV-1 capsid binding and restriction. J Virol. (2015) 89:3285–94. 10.1128/JVI.03730-14 25568212 PMC4337540

[60] Pedersen IM Cheng G Wieland S Volinia S Croce CM Chisari FV David M. Interferon modulation of cellular microRNAs as an antiviral mechanism. Nature. (2007) 449:919–22. 10.1038/nature06205 17943132 PMC2748825

[61] Swaminathan G Rossi F Sierra LJ Gupta A Navas-Martin S Martin-Garcia J. A role for microRNA-155 modulation in the anti-HIV-1 effects of Toll-like receptor 3 stimulation in macrophages. PLoS Pathog. (2012) 8:e1002937. 10.1371/journal.ppat.1002937 23028330 PMC3447756

[62] Pilakka-Kanthikeel S Nair PM. Interaction of drugs of abuse and microRNA with HIV: a brief review. Front Microbiol. (2015) 6:967. 10.3389/fmicb.2015.00967 26483757 PMC4586453

[63] Martinez-Nunez RT Louafi F Friedmann PS Sanchez-Elsner T. MicroRNA-155 modulates the pathogen binding ability of dendritic cells (DCs) by down-regulation of DC-specific intercellular adhesion molecule-3 grabbing non-integrin (DC-SIGN). J Biol Chem. (2009) 284:16334–42. 10.1074/jbc.M109.011601 19386588 PMC2713543

[64] Yamagata K Suzuki KN Sugiura H Kawashima N Okuyama S. Activation of an effector immediate-early gene arc by methamphetamine. Ann NY Acad Sci. (2000) 914:22–32. 10.1111/j.1749-6632.2000.tb05180.x 11085305

[65] Franchi S Moretti S Castelli M Lattuada D Scavullo C Panerai AE. Mu opioid receptor activation modulates Toll like receptor 4 in murine macrophages. Brain Behav Immun. (2012) 26:480–8. 10.1016/j.bbi.2011.12.010 22240038

[66] Hutchinson MR Zhang Y Shridhar M Evans JH Buchanan MM Zhao TX. Evidence that opioids may have toll-like receptor 4 and MD-2 effects. Brain Behav Immun. (2010) 24:83–95. 10.1016/j.bbi.2009.08.004 19679181 PMC2788078

[67] Akbari A Mosayebi G Samiei AR Ghazavi A. Methadone therapy modulate the dendritic cells of heroin addicts. Int Immunopharmacol. (2019) 66:330–5. 10.1016/j.intimp.2018.11.047 30521961

