# Supplementary material for: Methadone Inhibits Viral Restriction Factors and Facilitates HIV Infection in Macrophages
Source: Front Immunol. 2020 Jul 3;11:1253. doi: 10.3389/fimmu.2020.01253 (PMC7350609; doi:10.3389/fimmu.2020.01253)
Supplement: Supplementary file 1 [file Data_Sheet_1.doc]

**Figure.S1**

**
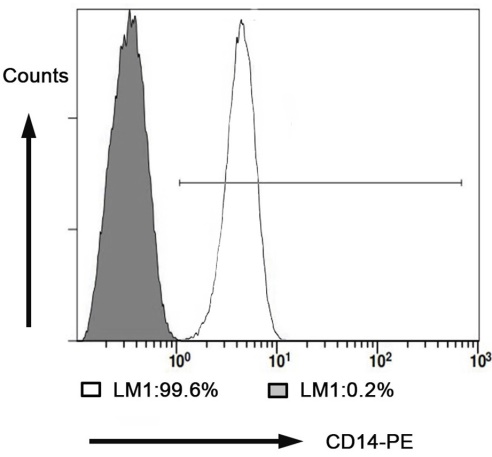
**

Figure S1. Monocytes were purified from peripheral blood and differentiated into macrophages after cultured for 7d. Cells were stained with fluorescence-conjugated anti-human CD14 antibody and analyzed for CD14 expression by flow cytometry.

**Figure.S2**

Figure S2. Methadone enhanced the expression of CCR5 in macrophages. Naltrexone (Nalt, 1 μM) was added to macrophages cultures for 1 h prior to methadone (MD, 1 μM)) treatment and macrophages were collected at 24 h post treatment. Total cellular RNA and protein were collected from macrophages. The expression of CCR5 (A) at mRNA level and protein level (B) were detected by real-time RT-PCR and Western blot, respectively. The results are presented as means ± standard deviations obtained from three independent experiments. The western blot is representative of three independent experiments (*, p＜0.05).

**Figure.S3**

**
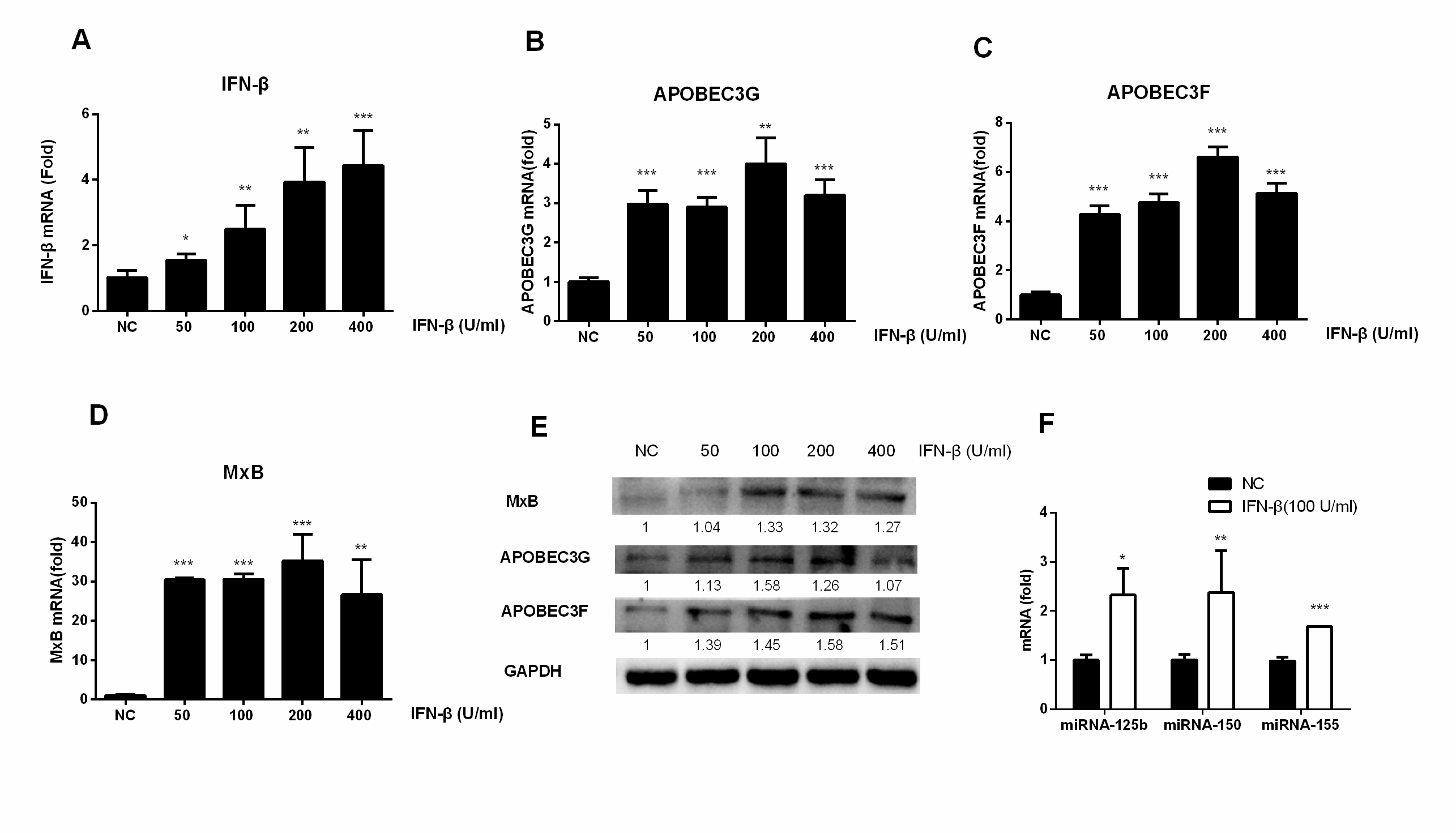
**

Figure S3. IFN-β treatment induced the expression of APOBEC3G, APOBEC3F, MxB and miRNAs. Macrophages were treated with IFN-β at indicated concentrations (0, 50, 100, 200, 400 U/ml) for 24 h. Total cellular RNA extracted from cells was collected for APOBEC3G, APOBEC3F, MxB and miRNAs (miRNA-125b, miRNA-150 and miRNA-155) gene by real-time RT-PCR (A-D,F) and the protein collected at 24 h was analyzed by Western blot (E). The results are presented as means ± standard deviations obtained from three independent experiments (***, p＜0.001; **, p＜0.01; *, p＜0.05).
